# Green, Sustainable Architectural Bamboo with High Light Transmission and Excellent Electromagnetic Shielding as a Candidate for Energy-Saving Buildings

**DOI:** 10.1007/s40820-022-00982-7

**Published:** 2022-12-10

**Authors:** Jing Wang, Xinyu Wu, Yajing Wang, Weiying Zhao, Yue Zhao, Ming Zhou, Yan Wu, Guangbin Ji

**Affiliations:** 1 College of Furnishings and Industrial Design, Nanjing Forestry University, Nanjing, 210037 People’s Republic of China; 2 College of Materials Science and Technology, Nanjing University of Aeronautics and Astronautics, Nanjing, 211100 People’s Republic of China; 3 College of Materials Science and Engineering, Nanjing Forestry University, Nanjing, 210037 People’s Republic of China; 4 State Key Laboratory for Modification of Chemical Fibers and Polymer Materials, Center for Advanced Low-Dimension Materials, College of Material Science and Engineering, Donghua University, Shanghai, 201620 People’s Republic of China

**Keywords:** Electromagnetic interference shielding, Biomass material, Transmittance, Energy-saving, Bamboo

## Abstract

Currently, light-transmitting, energy-saving, and electromagnetic shielding materials are essential for reducing indoor energy consumption and improving the electromagnetic environment. Here, we developed a cellulose composite with excellent optical transmittance that retained the natural shape and fiber structure of bamboo. The modified whole bamboo possessed an impressive optical transmittance of approximately 60% at 6.23 mm, illuminance of 1000 luminance (lux), water absorption stability (mass change rate less than 4%), longitudinal tensile strength (46.40 MPa), and surface properties (80.2 HD). These were attributed to not only the retention of the natural circular hollow structure of the bamboo rod on the macro, but also the complete bamboo fiber skeleton template impregnated with UV resin on the micro. Moreover, a multilayered device consisting of translucent whole bamboo, transparent bamboo sheets, and electromagnetic shielding film exhibited remarkable heat insulation and heat preservation performance as well as an electromagnetic shielding performance of 46.3 dB. The impressive optical transmittance, mechanical properties, thermal performance, and electromagnetic shielding abilities combined with the renewable and sustainable nature, as well as the fast and efficient manufacturing process, make this bamboo composite material suitable for effective application in transparent, energy-saving, and electromagnetic shielding buildings.

**Supplementary Information:**

The online version contains supplementary material available at 10.1007/s40820-022-00982-7.

## Highlights

- The whole bamboo mere made directly into cellulose composites, which greatly reduced massive energy consumption from the mechanical pre-processing process.
- The wall thickness of transparent whole bamboo can reach 6.23 mm.
- The modified whole bamboo possesses excellent optical transmittance of about 60% at 6.23 mm, the illuminance of 1000 luminance (lux), water absorption stability (the mass change rate less than 4%), longitudinal tensile strength (46.40 MPa), and surface properties (80.2 HD).
- The combination of excellent transmittance, mechanical properties, surface properties, thermal performance, and electromagnetic shielding properties makes this composite attractive in areas such as transparent, energy-saving, and electromagnetic shielding buildings.

**Supplementary Information:**

The online version contains supplementary material available at 10.1007/s40820-022-00982-7.

## Graphical abstract

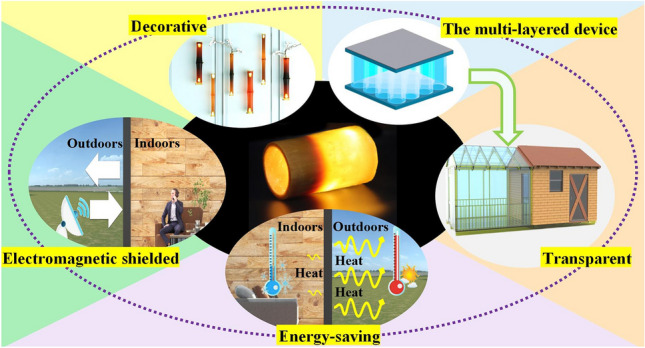

## Introduction

Currently, the development of green and energy-saving buildings to cope with energy consumption and achieve the goal of carbon neutrality at an early date is an important research topic [1–5]. Biomass materials not only retain their ecological characteristics, but also have the properties of regeneration, natural degradation, aesthetics, and regulation of the indoor environment, as well as low energy consumption and carbon sequestration, which have a positive impact on reducing CO_2_ emissions and mitigating climate warming [6–11]. Wood, bamboo, and other similar products are considered the raw materials for buildings [11–14]. Among them, wood-based transparent composites are a research trend for green and energy-saving buildings, such as transparent wood [15–17], bamboo [18, 19], and wheat straw [20]. Owing to their adjustable light transmittance and haze, light weight, low thermal conductivity, and good mechanical properties, they can provide a uniform and comfortable lighting environment and maintain room temperature (RT), which is advantageous in the field of energy-saving buildings [21–24]. The preparation of transparent biomass materials is mostly divided into two steps: removal of chromogenic groups and impregnation of a polymer with the same refractive index as that of the cellulose template [25–29]. In our previous work [30–32], an acid sodium chlorite solution was one of the most commonly used solutions for lignin removal [33–35]. Acid sodium chlorite solution selectively removes lignin without damaging cellulose and hemicellulose. However, due to the cost and harmful emissions, sodium chlorite method is not suitable for large-scale industrial delignification. Consequently, other environmentally friendly and efficient delignification methods have been proposed. Burgert et al. [36] proposed a method to remove lignin from wood using hydrogen peroxide (H_2_O_2_) and glacial acetic acid in 2018. Li et al. [37] reported a green and versatile method for steam-modified delignification using H_2_O_2_ and acetic acid to remove more lignin from wood in 2019. In past attempts, H_2_O_2_ and glacial acetic acid were successfully applied only to spruce and balsa wood with a low density (~ 0.4 g cm^−3^). H_2_O_2_ and acetic acid could remove the chromophore groups of lignin while preserving the macrostructure of the wood. However, no studies have reported the use of this mixture for the removal of lignin from bamboo, especially from untreated round, straight, and hollow bamboo tubes. In this study, the density of the bamboo used was 0.76 g cm^−3^, which is close to twice that of spruce and balsa wood. Therefore, it is challenging to target the whole bamboo using this method for delignification. Furthermore, this delignification process could be extended to other biomass materials with high densities and low porosities.

As one of the most important biomass resources worldwide, bamboo is also a candidate for the preparation of transparent composites with the advantages of sustainability, rapid growth, and abundant resources [38–40]. Bamboo is a natural composite composed of a vascular bundle (fibers) embedded in parenchymal cells (basic organization) that function as a link [41–43]. It forms an optimized structure for the number of vascular bundles increasing from inner wall thickness to outer wall in the natural environment [44]. More time needs to be spent removing lignin and impregnating polymers from bamboo than from wood owing to its lower porosity. It is also challenging to prepare transparent bamboo of a certain thickness. To improve the thickness of transparent bamboo than in our previous study, we prepared transparent bamboo through multilayer superposition [45, 46]. Through delignification and impregnation of bamboo sheets with epoxy resin, five layers of transparent bamboo (less than 1.5 mm) achieved a transmittance of 63%. However, the thickness of the transparent bamboo was less than 2 mm.

Over the last several years, few attempts have been made to directly process bamboo into cellulose composites with desirable optical properties. In our previous study, transparent bamboo was used as a sheet or plate. Transparent bamboo sheets show tremendous prospective applications in ceilings, furniture veneer decorations, substitution for architectural glass, etc. Nevertheless, transparent bamboo sheets are limited by thicknesses and widths. The thickness and width of the transparent bamboo sheets do not exceed 2 mm and 300 × 500 mm^2^, respectively. Bamboo sheets cut using plywood are commonly adopted as raw materials for preparing transparent bamboo sheets. The complex and tedious pre-processing steps are not only labor-intensive, but also consume a large amount of non-renewable resources, such as fuel and electricity. However, whole bamboo can be processed directly into cellulose composites, which greatly reduces the need for mechanical pre-processing. This also implies a reduction in the consumption of non-renewable resources.

Although the rapid development of wireless communication technology and electrical equipment has increased convenience in people’s lives, problems such as electromagnetic interference and radiation, and electromagnetic leakage, have also increased [47–50]. The increasingly deteriorating electromagnetic environment not only endangers people’s health, but also interferes with the normal operation of all types of electronic equipment. Therefore, it is essential to study electromagnetic shielding materials used in buildings and homes [51]. To further explore the application potential of translucent whole bamboo materials in other fields, an electromagnetic shielding film covering was used. In this study, a film-like structure was selected to achieve electromagnetic shielding of the multilayered device, owing to the more homogeneous nature of the film layers, which ultimately achieved both optical transparency and effective electromagnetic shielding. Transparent conductive films include tin-doped indium oxide (ITO), F-doped SnO_2_ (FTO), Co-doped SnO_2_ (CTO), Al-doped ZnO (AZO), Ga-doped ZnO (GZO), CuS, PbO, and other transparent conductive films [52–55]. The ITO film is a transparent conductive oxide film with a light transmission of more than 85% in the visible range. Tin-doped indium oxide films have the advantage of high hardness, superior abrasion, and chemical resistance [56–59]. Combining a transparent biomass material with an ITO film will help to produce building and home materials with advantages such as light transmission, heat insulation, and electromagnetic shielding.

**Fig. 1:**
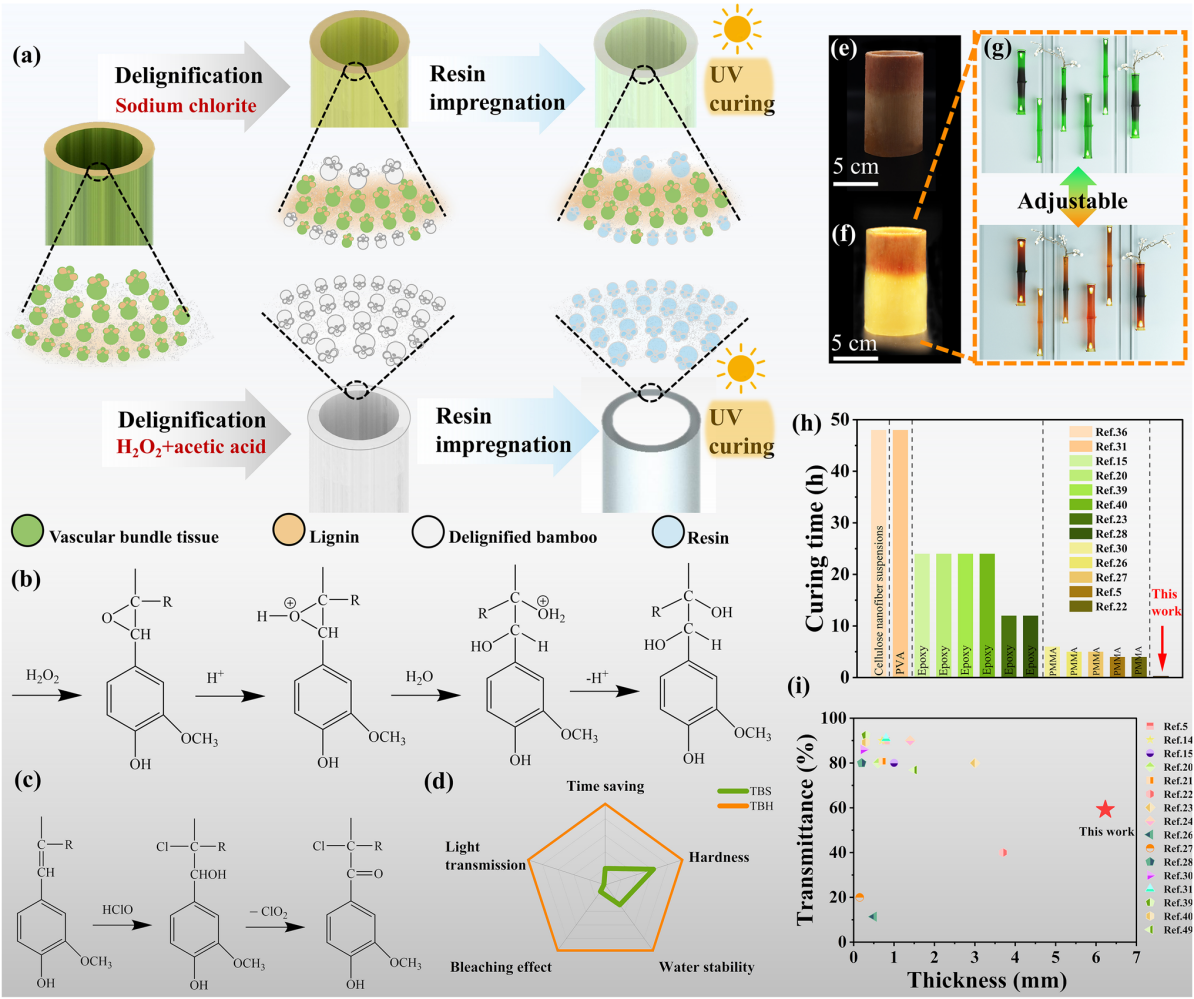
**a** Schematic illustration of the preparation of cellulose composites by different delignification methods. **b** Reaction of lignin in a mixed solution of hydrogen peroxide and acetic acid. **c** Reaction of lignin in acidic sodium chlorite solution. **d** Radar plot showing the comparison between TBS and TBH. **e** Macroscopic view of TBS. **f** Macroscopic view of TBH. **g** Applications of TBH in lighting materials. **h** Comparison of curing time of our work and other biomass transparent materials impregnated by different polymers. **i** Comparison of thickness of our work and other biomass transparent materials

To address the above challenges, herein, we developed a simple, fast, and efficient approach to process whole bamboo into a translucent cellulose composite material that retains its natural structure and good mechanical properties through delignification and impregnation. In this process, whole bamboo was delignified with a mixture of H_2_O_2_ and glacial acetic acid and impregnated with UV curing resin to obtain a translucent cellulose composite (TBH). For comparison, cellulose composites (TBS) were prepared by acid sodium chlorite delignification and impregnation with a UV curing resin (Fig. 1a). Sodium chlorite is prone to decomposing under acidic conditions. During the removal of lignin from bamboo by acidic sodium chlorite, chlorine dioxide radicals effectively and selectively oxidized olefin side chains and aromatic ring groups in lignin without damaging the cellulose structure (Fig. 1b). As shown in Fig. 1c, the oxidation of H_2_O_2_ under conditions was greatly enhanced [60]. Consequently, H_2_O_2_ reacted with acetic acid to produce peracetic acid, with which lignin reacted to form the trans-dihydroxylation addition product. This was converted to an epoxide intermediate following the electrophilic addition reaction mechanism. Using whole bamboo as a raw material was conducive to obtaining a complete cellulose skeleton. Furthermore, compared with TBS, TBH had a better bleaching effect, was less time-consuming, and possessed the optical properties of bamboo, with higher hardness and water absorption stability (Fig. 1d). As shown in Fig. 1e–f, TBH with optical properties has greater potential for application in the fields of lighting and decorative materials than TBS (Fig. 1g). Compared to other biomass transparent samples prepared by different polymer impregnation methods, the curing time of our samples was extraordinarily short, hence showing a remarkable potential for rapid processing (Fig. 1h). Moreover, in this work, the thickness of the samples reached 6.23 mm, showing a huge advantage in terms of thickness compared to previous biomass transparent materials (Fig. 1i). The samples were numbered for convenience in describing the sample characteristics, as shown in Table S1.

Translucent bamboo was further fabricated into a multilayered device. The multilayered device structure in our study is similar to that of a honeycomb board. The role of transparent bamboo sheets in multilayered devices is equivalent to that of a panel in a honeycomb sandwich structure. Similarly, the role of translucent bamboo joints in multilayered devices is equivalent to that of the core material in a honeycomb sandwich structure, providing light transmission, mechanical strength, stability, and aesthetic value. Tin-doped indium oxide films in this module provide the multilayered devices with electromagnetic shielding characteristics, highlighting its broad application in the field of electromagnetic shielding. Multilayered devices tend to be used in the field of architectural decorative materials, such as interior space partitions, merchandise displays, billboards, lighted ceilings, furniture decoration, and lighting design.

## Experimental Section

### Materials and Chemicals

Five-year-old Moso bamboo (*Phyllostachys heterocycla*) with 13% water content, obtained from Yifeng County, Jiangxi Province, China, was collected from the original bamboo samples. The average height of the whole bamboo after removing the green part was 103.47 mm, the average wall thickness was 5.51 mm, the average diameter of the outer cylinder was 54.50 mm, the air-dry density was 0.76 g cm^−3^, and the absolute dry density was 0.67 g cm^−3^. Analytical grade glacial acetic acid, 30% H_2_O_2_, and absolute ethanol were purchased from Nanjing Chemical Reagent Co., Ltd. UV curing resin (model a185721007), mainly composed of epoxy acrylate and polyurethane acrylate, was provided by China Huzhou Polymer Materials Co. Ltd. All the above chemicals were used directly in the laboratory without other purification treatments. An ITO film with a resistivity of 0.9 ohms was purchased from Frontier Radiation Protection Technology Co. Ltd.

### Pre-processing

Whole bamboo samples, after removing the outer green part, were dried in an oven at 103 °C, for 12 h.

### Preparation of Delignified Bamboo Templates Using Glacial Acetic Acid and Hydrogen Peroxide

A delignification solution with 50 wt% glacial acetic acid and 50 wt% 30% H_2_O_2_ was prepared. The whole bamboo samples were soaked in the prepared solution and maintained at 80 °C for several hours until the sample in the delignification solution became white and light transparent. The bamboo cellulose templates were then removed and rinsed with distilled water several times to wash out the remaining chemical reagents. Finally, the samples were placed in absolute ethanol to obtain delignified bamboo templates.

### Preparation of Delignified Bamboo Templates by Acid Sodium Chlorite

A 5 wt% solution of sodium chlorite was prepared, and an appropriate amount of glacial acetic acid was dropped into it; then, the whole bamboo samples were placed among the solutions after adjusting the solution pH to 4.6, which was then heated in a water bath at 80 °C for 12 h. After the samples were removed, they were rinsed several times with distilled water to wash away the sodium chlorite drug product remaining in the samples. Finally, the samples were placed in absolute ethanol to obtain the bamboo templates.

### Impregnation with UV Curing Resin

The samples were vacuum impregnated in UV curing resin for 48 h. A UV aging device was used to cure the samples with UV light for 15 min to obtain cellulose composites treated with acid sodium chlorite, translucent cellulose composites treated with H_2_O_2_, and glacial acetic acid.

### Preparation of the Multilayered Device

To explore the application potential of translucent bamboo in architectural decoration and electromagnetic shielding, UV resin was used to bond the translucent whole bamboo, transparent bamboo sheets, and ITO film together to form a multilayered device.

### Characterization

The chemical groups in all samples were characterized and analyzed using Fourier transform infrared spectroscopy (FTIR, ERTEX 80 V, Germany). The samples were analyzed using an X-ray single-crystal diffractometer (XRD, Bruker D8 Venture, Germany). The relative lignin content in the samples was measured using the method described by the National Renewable Energy Laboratory. The cross sections and longitudinal sections of the samples were investigated using scanning electron microscopy (SEM, quanta200, FEI, USA). A UV visible photometer (U3900, HITACHI, Japan) was used to measure the optical transmittance of the samples under visible light at wavelengths of 380–780 nm. The L*a*b coordinates of the samples were recorded using an RM200 color tester. The ASAP2020 automatic specific surface area and pore size distribution instrument was used to measure the specific surface area of samples. A TES-1330A digital illuminometer was used to measure the illumination of the samples at various distances. Longitudinal tensile tests were carried out on the samples using an AG-IC precision electromechanical testing machine with a tensile rate of 3 mm min^−1^ and a maximum load force of 10,000 N. The surface hardness values of the samples were measured and recorded using an LX-D Shao rubber durometer. The thermal degradation of samples from 0–800 °C was tested using a thermogravimetry and synchronous thermal analyzer (TGA, METTLER Toledo TGA/DSC1, Sweden) under a nitrogen atmosphere at a heating rate of 10 °C min^−1^. The mass of the samples before and after water absorption was recorded after being placed in distilled water solution at RT (25 °C) for more than 25 h, and the change ratio of the mass was calculated. The infrared emissivity of the multilayered device was measured using a dual-band emissivity meter (IR-2, Shanghai Chengbo Optoelectronic Technology Co. LTD) at 3 ~ 5 and 8 ~ 14 μm. The multilayered device and extruded polystyrene thermal insulation (XPS) board were placed on the heating gasket, and infrared thermal images of the multilayered device and XPS board were captured using an infrared camera (FOTRIC 227S). A temperature recorder was used to record the changes in the temperature of the uppermost layer of the multilayered device and the XPS board every second within 5 min. The vector network analyzer N5244A of Agilent and the waveguide method were used to test the shielding effectiveness (SE) of the samples in the X-band (8.2 GHz ~ 12.4 GHz) at RT. More detailed formulae can be found in the supplementary material.

## Results and Discussion

**Fig. 2:**
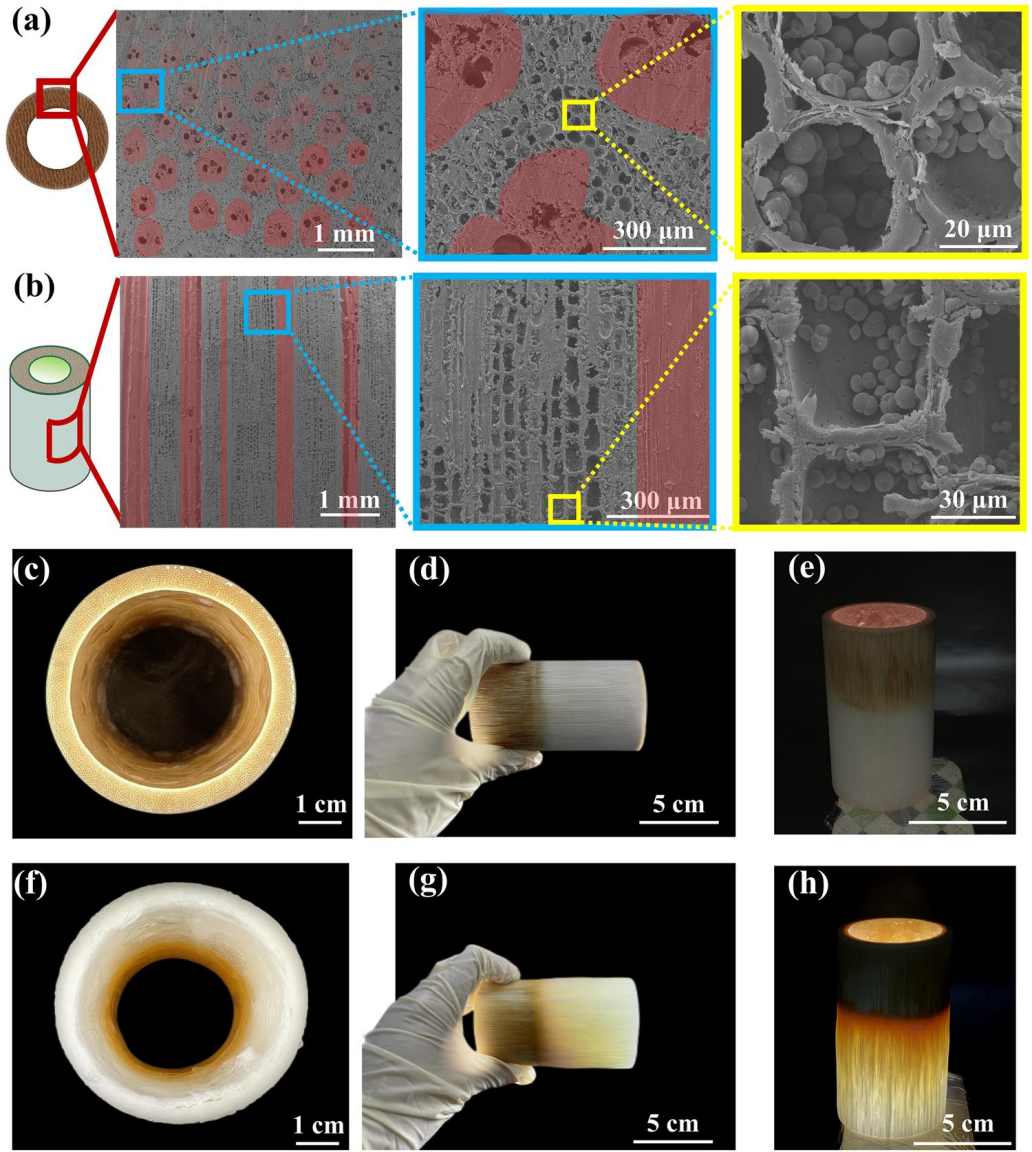
**a** Cross-sectional SEM images of OB. **b** SEM images of the longitudinal section of OB. **c** Photograph of the cross section of S12. **d** Macroscopic view of S12. **e** Macroscopic view of S12 under the light. **f** Photograph of the cross section of H12. **g** Photograph of H12. **h** Photograph of H12 under the light

Bamboo is a composite material that contains reinforcing component fibers and matrix cells. In Fig. 2a–b, the vascular bundles aligned along the axis contributed the most to the mechanical properties of bamboo (marked in red). The vascular bundles mainly consisted of fibrocytes and ducts, and the basic tissue consisted of numerous parenchymal cells. These cells were divided into reticular parenchyma cells and polygonal parenchyma cells surrounding the fibers. Generally, the longitudinal length and transverse width of parenchyma cells are 300 ~ 1200 and 15 ~ 200 μm, respectively [38]. The vascular bundles and basic tissues were unevenly distributed in the thickness direction of the bamboo wall; the closer the outer side of the bamboo wall, the smaller the body shape of the vascular bundle, the denser the distribution, and the lower the number of basic tissues. Many spherical or hemispherical starch grains were present in OB thin-walled cells.

To obtain whole bamboo with optical properties, lignin was first removed from the bamboo. Only the outer and inner walls of the whole bamboo directly contacted the solution and became white following treatment in acidic sodium chlorite solution for 12 h (Fig. 2c). There was no obvious change after whole bamboo was treated in acidic sodium chlorite solution for 12 h (Fig. S1a). As shown in Fig. 2d–e, light could not pass through the entire bamboo treated with sodium chlorite for 12 h. In contrast, the whole bamboo gradually turned white in a mixed solution of H_2_O_2_ and acetic acid (Fig. S1b). Bamboo treated with different delignification methods exhibited different chromaticity changes (Figs. S2–S3). Compared to the delignified samples treated with acid sodium chlorite without color change, the delignified samples treated with the H_2_O_2_ and acetic acid showed a significant increase in brightness with increasing treatment time. In alkaline media, H_2_O_2_ can break and dissolve the benzene rings and side chains of lignin, thus destroying the chromophoric group in lignin and achieving bleaching. Figure 2f–h shows that the part soaked in the solution completely turned white after treatment for 12 h, and light could penetrate the part after delignification treatment (Movie S1).

**Fig. 3:**
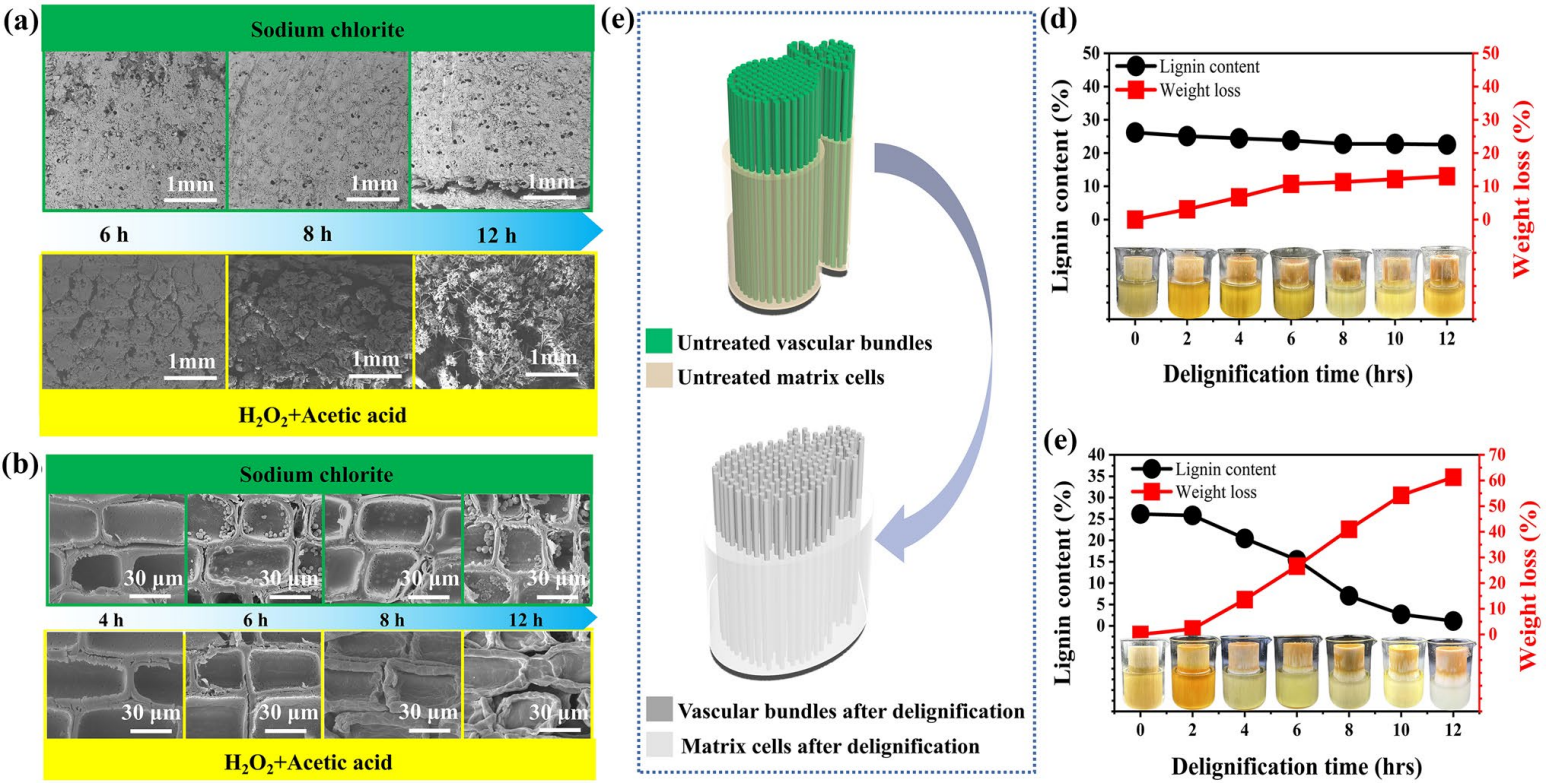
**a** Cross-sectional SEM images of the whole bamboo treated with different delignification methods. **b** Longitudinal-sectional SEM images of parenchyma cells of the whole bamboo treated with different delignification methods. **c** Schematic diagram of the variation of bamboo fiber bundles and matrix cells treated with a mixture of hydrogen peroxide and acetic acid. **d** Variation of weight loss rate and relative lignin content of samples in acidic sodium chlorite solution. **e** Variation of weight loss rate and relative lignin content of samples in the mixed solution of hydrogen peroxide and acetic acid

In addition to macroscopic changes, the microstructure of bamboo also changed during chemical treatment. Figure 3a–b shows changes in the microstructure of bamboo in the cross- and longitudinal sections directions over time in the acidic sodium chlorite solution. The bamboo structure remained relatively intact, without serious deformation. As the delignification time increased and the cell walls gradually became thinner; the parenchyma cells were not severely deformed, and the intercellular spaces were not enlarged. This indicates that the lignin of the whole bamboo could not be completely removed by an acidic sodium chlorite solution. Simultaneously, no more pores were generated, which was unfavorable for resin impregnation. We also found that the delignification effect of whole bamboo in the mixed solution was more obvious under the same treatment time and temperature conditions (Fig. 3c).

Changes in the weight loss rate and lignin content in the acidic sodium chlorite solution are shown in Fig. 3d. The relative lignin content decreased from 26.17 to 22.55% after 12 h. The rate of bamboo weight loss increased slowly over time, reaching 13.01% after 12 h. Apparently, the oxidizing performance of sodium chlorite was not sufficient to treat the whole bamboo. Therefore, the lignin was not completely removed from the whole bamboo. We observed that during the treatment process, lignin content was greatly reduced to 1.13% (Fig. 3e). The rate of bamboo weight loss increased significantly during the process of greatly reducing lignin content. After 12 h, the bamboo weight loss rate reached 61.27%, indicating that the lignin was successfully removed following H_2_O_2_ and acetic acid treatment. Compared to OB, the specific surface area of H12 increased from 0.1062 to 0.4292 m^2^ g^−1^. The removal of lignin means the disappearance of the “binder” (connecting cellulose and hemicellulose), leading to the appearance of more pores to be filled with UV resin. Simultaneously, the relative cellulose content of the bamboo increased from 39.5 to 69.7% over the course of the treatment, possibly due to a significant decrease in the lignin content. Additionally, the relative hemicellulose content of the bamboo did not change significantly (Fig. S4).

**Fig. 4:**
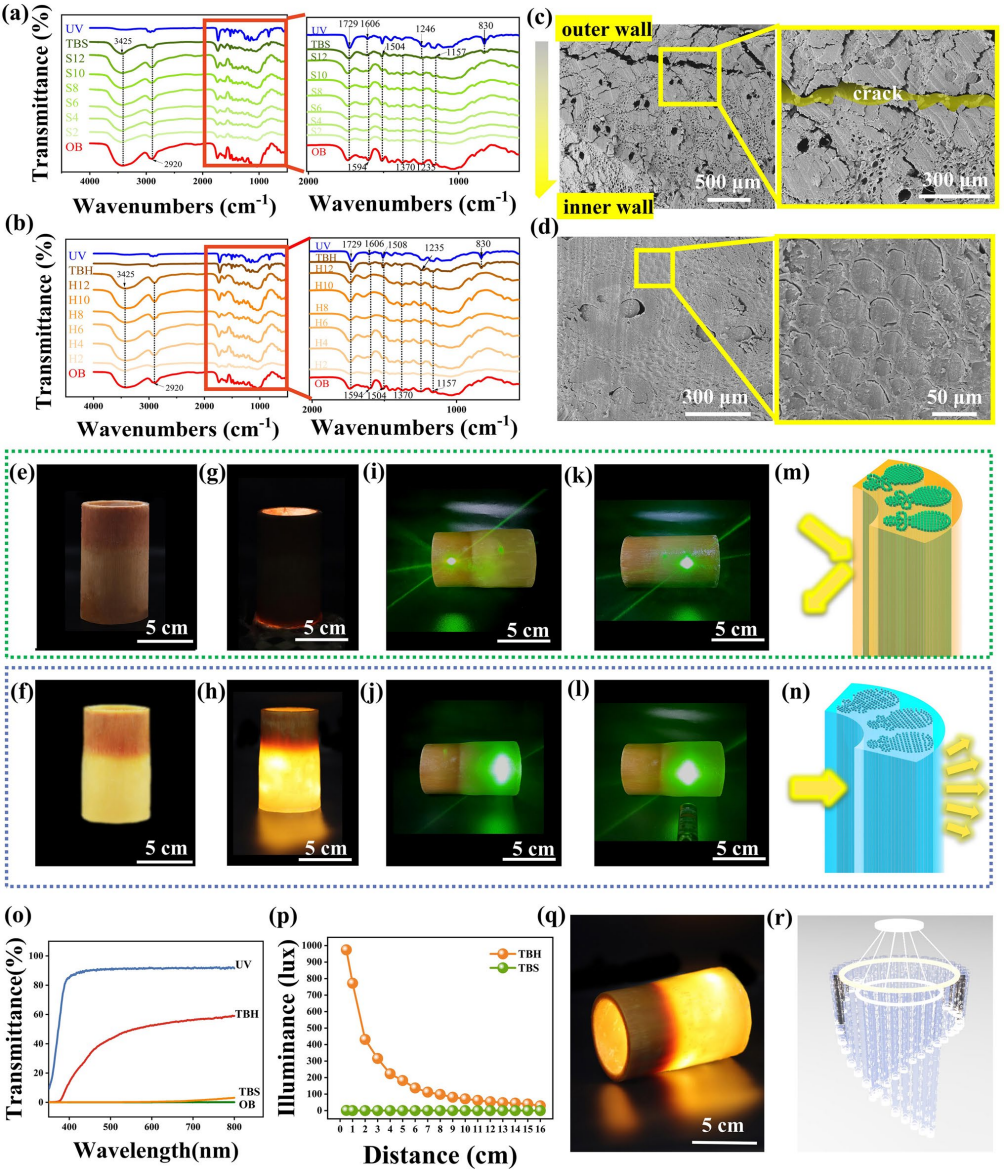
**a** FTIR curves of OB, S2, S4, S6, S8, S10, S12, TBS, and UV resin. **b** FTIR curves of OB, H2, H4, H6, H8, H10, H12, TBH, and UV resin. **c** Cross-sectional SEM images of TBS. **d** Cross-sectional SEM images of TBH. **e** Digital image of TBS. **f** Digital image of TBH. **g** Digital image of TBS with LED lights installed. **h** Digital image of TBH with LED lights installed. **i** Laser irradiation of untreated bamboo. **j** Laser irradiation of TBH. **k** Laser irradiation of TBS. **l** Laser irradiation of TBH. **m** Schematic diagram of the reflection of the light beam on the surface of untreated bamboo. **n** Schematic diagram of the light beam passing through TBH. **o** Light transmission of OB, TBS, TBH, and UV resin. **p** Illumination change diagram of TBH and TBS. **q** Macroscopic view of TBH, **r** A rendering of a decorative lighting fixture designed by translucent whole bamboo

Figure 4a shows that the OB sample at 3425 cm^−1^ (hydroxyl stretching vibration), 2920 cm^−1^ (methyl, methylene, and methine stretching vibration), 1729 cm^−1^ (stretching vibration peak of a hemicellulose acetyl group), 1594 cm^−1^ (C = O stretching vibration peak), 1504 cm^−1^ (lignin aromatic ring skeleton vibration), 1370 cm^−1^ (aromatic ring C-H stretching vibration), 1235 cm^−1^ (lilac ring C-O stretching vibration), and 1157 cm^−1^ (C–O–C asymmetric vibration peak) produced characteristic peaks, consistent with that of previous research [61–63]. However, the vibration peaks at these wavelengths after bamboo delignification by acid sodium chlorite alone did not change significantly (Fig. 4a-b), which means that the lignin content did not change remarkably. In contrast, over time, the characteristic vibrational peaks at 1594 and 1504 cm^−1^ of the delignified samples after delignification with the mixed solution gradually weakened until they disappeared. There were no C = O or phenolic hydroxyl groups in the lignin of the delignified samples. Therefore, lignin was successfully removed after this delignification treatment. Notably, the XRD curves of all delignified samples were similar to those of OB, indicating that neither sodium chlorite, H_2_O_2_, nor acetic acid affected the crystalline structure of cellulose in bamboo (Fig. S5). Additionally, the vibrational peaks at other wavelengths of the delignified bamboo template samples did not change significantly, indicating that large amounts of cellulose and hemicellulose were retained in the delignified bamboo templates. This suggests that the microstructure and hierarchical arrangement of the bamboo were well preserved throughout the manufacturing process.

To change the transmission path of light and reduce light scattering, H12 and S12 were impregnated with a UV resin with a refractive index matching that of the bamboo fiber templates. Subsequently, TBS and TBH produced characteristic peaks at wavelengths of 1729 cm^−1^ (the vibrational peak of conjugated C = O), 1606 cm^−1^ (the vibrational band of the asymmetric benzene ring skeleton), and 830 cm^−1^ (the plane deformation peak of the para-substituted benzene ring = CH). Notably, the UV resin could only impregnate the outermost layer of TBS, while the other parts remained unfilled (Fig. 4c). Combined with the SEM images (Fig. 3a), only the outermost and innermost walls in contact with the delignification solution were delignified. Moreover, no more pores appeared in the rest of the bamboo, which was not conducive to UV resin filling. Although the outermost layer of the bamboo wall was filled with UV resin, there were many gaps between the cells. Furthermore, there was an obvious crack between the middle structure and the outermost layer that has been impregnated with the UV resin. Different degrees of light scattering occurred in these empty cell cavities and gaps, which may have prevented TBS from being translucent. Figure 4d indicates that the pore cavities of the bamboo were filled with the UV resin. The UV resin successfully impregnated the delignified bamboo template that was treated with the mixed solution and was compatible with the bamboo cell matrix.

After impregnating the UV resin, the lightness of the surface of the sample decreased, i.e., the samples became darker. However, TBH presented a more aesthetically valuable amber texture. Translucent cellulose composite is a light-transmitting material, while TBS is an opaque material (Fig. 4e–h). Filling them with a UV resin with a refractive index similar to that of the bamboo cell wall can effectively reduce their light refraction. Furthermore, compared to other biomass transparent samples prepared by different polymer impregnation methods, the curing time of TBH was extraordinarily short, showing a remarkable potential for rapid processing (Table S2). In contrast to TBS, which is filled with pores, TBH is a more homogeneous material because few pores exist between the cells and interconnected adjacent cell walls. Therefore, TBH exhibits superior optical properties to TBS.

Figure 4i–l shows photographs of the laser-irradiated samples. When the laser beam irradiated the surface of untreated bamboo or TBS, the path of the beam did not change significantly, and obvious light reflection occurred. In contrast, when the laser beam irradiated the surface of the TBH, the beam developed a light scattering phenomenon on the surface of the sample (Fig. 4m–n). We determined the total transmittance of OB, UV resin, TBH, and TBS in the visible light range at wavelengths of 380 ~ 800 nm (Fig. 4o). Notably, OB and TBS had almost no optical transmittance in the visible light range. A 6.23-mm-thick TBH showed a certain optical transmittance, with a maximum transmittance of 59.2%. In our previous study, the light transmittance of 2.9-mm-thick transparent bamboo designed by multilayer superimposition was 53.7% [46]. However, when the thickness of the TBH was doubled, the light transmittance also increased by 10%. Moreover, the whole bamboo could be processed directly into cellulose composites, which greatly reduced the massive energy consumption of the mechanical pre-processing steps and helped to retain the complete cellulose skeleton structure of the whole bamboo. In addition, the translucent whole bamboo could have a wall thickness of up to 6.23 mm, which is superior to that of most reported works (Table S2). Figure 4p shows that TBH had a higher luminosity than TBS; the maximum luminosity of TBH could be close to 1000 lux, while that of TBS was less than 2 lux This suggests that TBH can be a candidate material for providing lighting and has the potential to be used in home decoration and lighting applications (Fig. 4q-r).

**Fig. 5:**
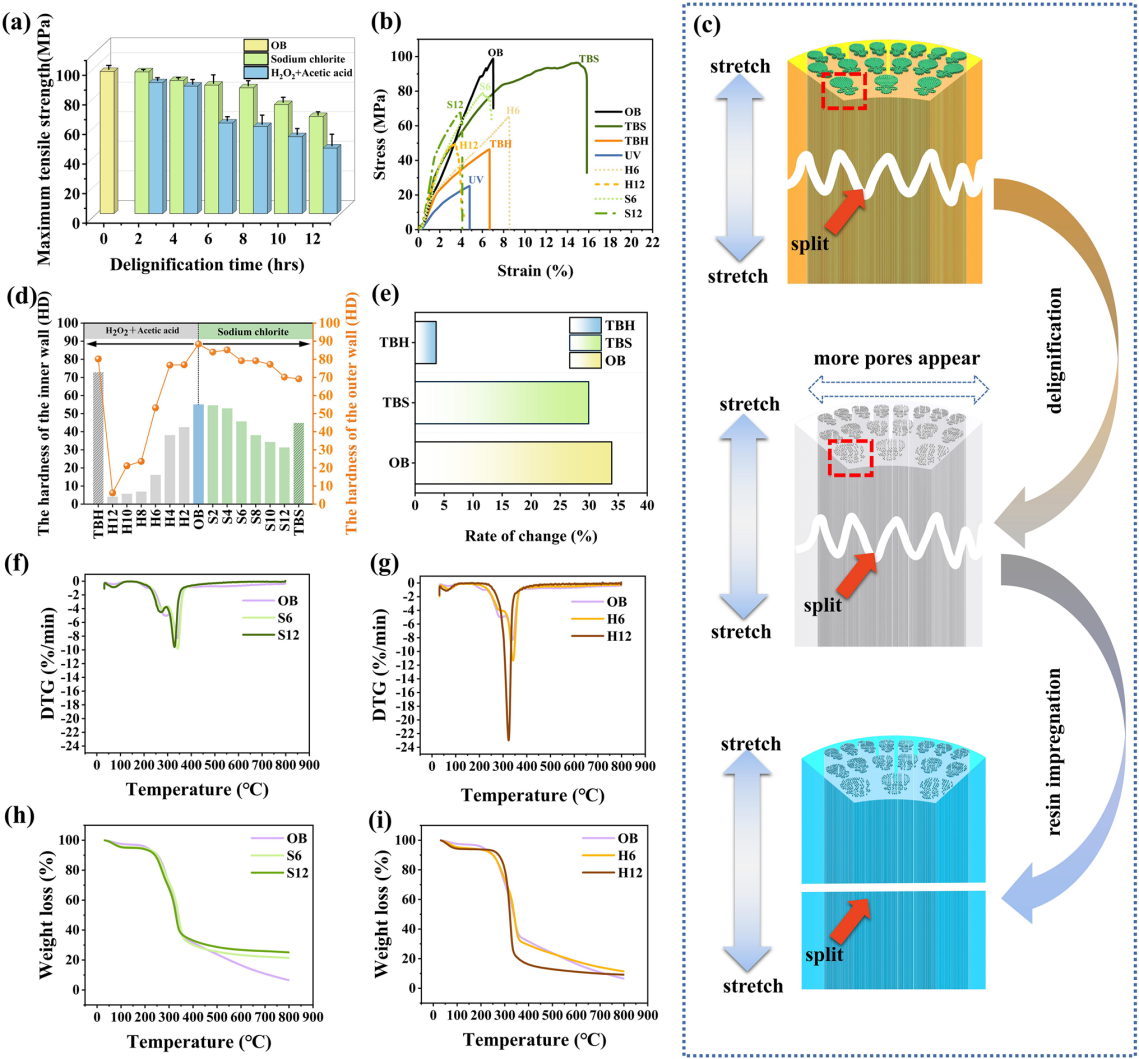
**a** Maximum tensile strength of OB and delignified bamboo. **b** Stress–strain curves of OB, TBS, TBH, UV, H6, H12, S6, and S12. **c** Schematic diagram of fracture of different samples when being stretched. **d** Surface hardness of the samples. **e** Mass change rate after water saturation of OB, TBS, and TBH. **f** DTG curves of OB, S6, and S12. **g** DTG curves of OB, H6, and H12. **h** TG curves of OB, S6, and S12. **i** TG curves of OB, H6, and H12

The strength of bamboo is the result of joint loading of fiber bundles and parenchyma cells. From the point of view of fiber-reinforced composites, fiber bundles in natural bamboo structures can be regarded as fiber-reinforced components and parenchyma cells as a matrix [64]. Therefore, bamboo, as a natural organic macromolecular polymer, has good tensile properties. The maximum tensile strength of OB was 98.81 MPa along the fiber direction (Fig. 5a). The two different delignification methods undoubtedly had different effects on the tensile properties of bamboo. Among them, the mixed solution had the greatest influence on the tensile properties of bamboo. Note that the maximum tensile strengths of S12 and H12 are 67.58 and 47.97 MPa, respectively, indicating that that of bamboo treated with sodium chlorite method and H_2_O_2_ as well as acetic acid slightly decreased. This may be primarily because H_2_O_2_ and acetic acid solution remove a large amount of lignin, leading to the destruction of the bamboo structure. Two main factors affect the tensile strength of transparent bamboo. The first is lignin removal rate from bamboo material. As a complex amorphous substance, lignin is connected to cellulose and hemicellulose in the form of hydrogen and covalent bonds in bamboo to provide structural support [72]. The tensile strength of TBH was higher than that of other biomass transparent materials impregnated with other polymers (Table S3). As shown in Fig. 5b, after the transparency treatment, the maximum tensile strengths of TBH and TBS were 46.40 and 82.46 MPa, respectively, implying that the mechanical properties of the transparent treated samples were directly related to the lignin content. After delignification, the bamboo cell walls were destroyed, leading to a remarkable decrease in the mechanical properties. Moreover, the polymer impregnated into the bamboo template is a key factor influencing the maximum tensile strength of composite materials. The interfacial compatibility of polymer and cellulose template directly affects the mechanical properties of composites. The UV resin used in this study had the advantages of low viscosity, UV curing, and fast curing speed. Meanwhile, it also has the disadvantage of low maximum tensile strength and high brittleness (Fig. 5c). Therefore, the selection of the resin also plays a crucial role in the tensile properties of transparent biomass materials.

Bamboo is mainly composed of vascular bundles and parenchyma cells. The outer layer of bamboo has more vascular bundles than the inner layer, and fewer parenchyma cells than the inner layer. Parenchyma cells are soft and their density is lower than that of the fibers. Therefore, the surface hardness of the bamboo outer wall is greater than that of the inner wall. As shown in Fig. 5d, the surface hardness of the inner and outer walls of bamboo could be as high as 55 and 88.4 HD, respectively. Compared to bamboo treated with the acid sodium chlorite method, the surface hardness of the inner and outer walls of bamboo treated with the mixed solution decreased significantly with increase in treatment time. After 12 h, the surface hardness of the inner and outer walls of bamboo was 4 and 6.2 HD, respectively, indicating that bamboo changed from being dense to loose after treatment. After the transparency treatment, the surface hardness of the inner and outer walls of TBS was 44.8 and 69.2 HD, respectively, whereas those of TBH were 72.8 and 80.2 HD, respectively. The difference in hardness between the outer and inner walls of TBS remained large, while that of TBH was small, reinforcing that TBH is a homogeneous material with a better surface damage resistance than TBS. The dimensional stability of the samples was measured to explore the potential of TBH as an architectural material. Interestingly, the rate of mass change after reaching the saturation point of water absorption varied from sample to sample (Fig. 5e). The rate of mass change of OB, TBS, and TBH was approximately 33.89%, 29.91%, and less than 4%, respectively. Owing to their porous structures, with incomplete delignification and unsuccessful resin impregnation, OB and TBS absorbed a large amount of water. In contrast, the pores of TBH were completely filled with UV resin, which greatly reduced water absorption and improved dimensional stability.

As shown in Fig. 5f–g, OB experienced three different weight loss stages at 30–100, 200–350, and 315–400 °C, due to the evaporation of water and decomposition of hemicellulose, lignin, and cellulose, respectively. Lignin is a complex aromatic polymer, and the pyrolysis of lignin occurs almost throughout the entire process, but the main degradation of lignin occurs above 350 °C [65, 66]. S6, S12, H6, and H12 treated by two different delignification methods also exhibited similar weight loss curves to OB (Fig. 5h–i). However, the weight loss rate of H12 at approximately 315 °C was much higher than that of other delignified samples, which may be because the lignin in H12 has been basically removed without the participation of lignin in the pyrolysis process. The weight loss rate of TBH was significantly lower than that of H12. During the weight loss of TBH, a weak peak appeared at ~ 370 °C and a strong peak at ~ 410 °C, corresponding to the two-step thermal decomposition. The first step is the breakage of chemical bonds with lower bond energies (-C–C-, -O–H, etc.) in the UV resin structure. The second step involves the breaking of chemical bonds with a higher bond energy (-COOH, etc.), which is similar to the weight loss curve of the UV resin (Fig. S6). Figure S7 shows that the residual weight of TBH was higher than that of TBS before the temperature rose to 400 °C; therefore, the thermal stability of TBH was higher than that of TBS, probably due to the successful impregnation of UV resin.

**Fig. 6:**
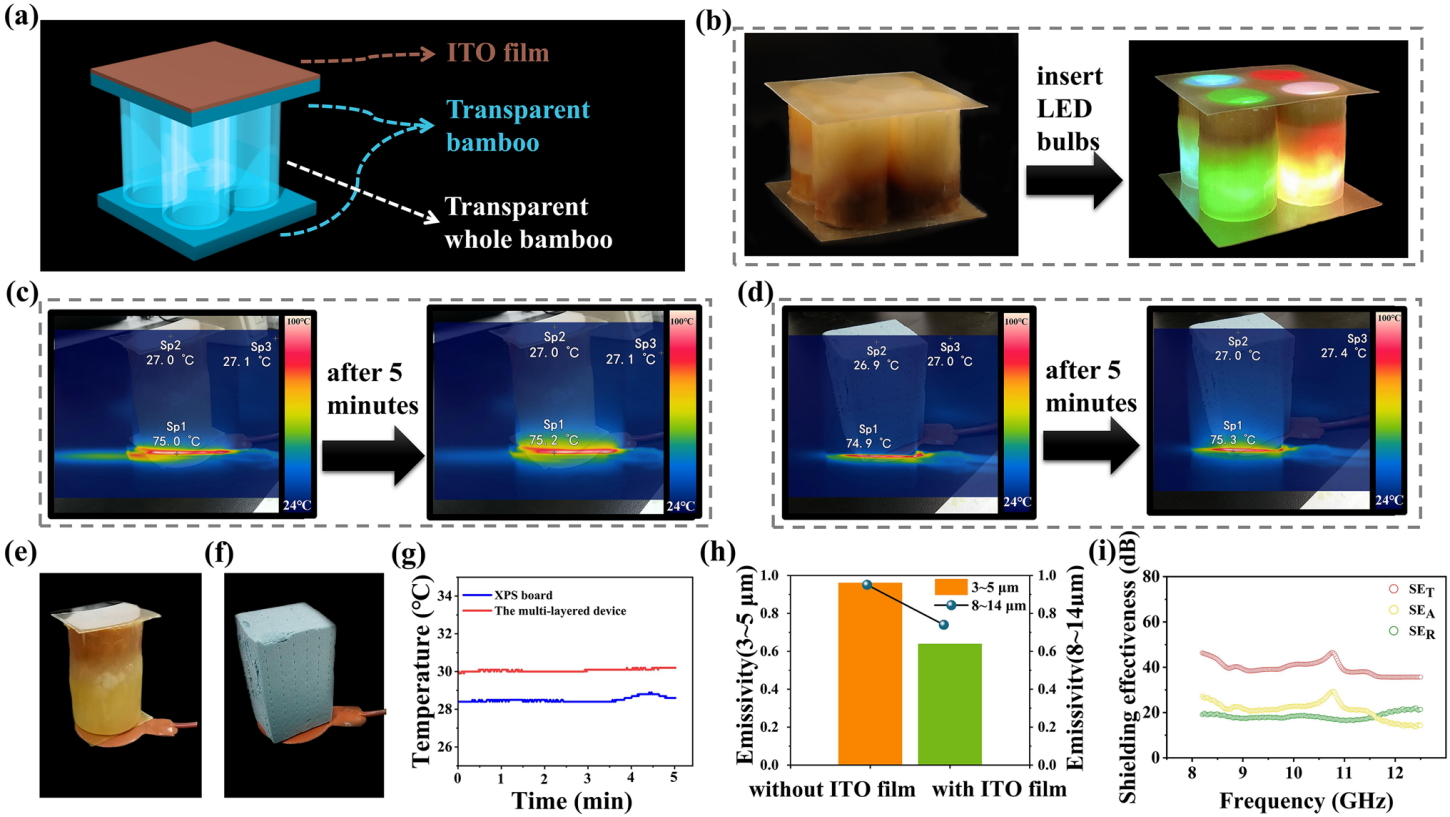
**a** Schematic diagram of the multilayered device composed of translucent whole bamboo, transparent bamboo, and ITO film. **b** Comparison before and after installing LED light bulbs in the multilayered device. **c** Infrared thermal image of the multilayered device before and after heating. **d** Infrared thermal image of XPS insulation board before and after heating. **e** Photograph of the multilayered device on the heating platform. **f** Photograph of the XPS insulation board on the heating platform. **g** Temperature variation diagram of the multilayered device and XPS insulation board on the heating pad. **h** Infrared emissivity at 3 ~ 5 μm and 8 ~ 14 μm of the multilayered device. **i** Shielding effectiveness of the multilayered device

A multilayered device consisting of translucent whole bamboo, transparent bamboo sheets, and an ITO film was used to demonstrate the practicability of this composite material in the fields of construction, housing, and furniture (Fig. 6a). After small LED bulbs of different colors were inserted into the multilayered device, it exhibited good light transmittance, high aesthetic value, which showed its potential for use as decorative and lighting materials (Fig. 6b and Movie S2). Figure 6c–d shows the infrared thermal images of the multilayered device and commercial insulation board, respectively. After heating the bottom of the sample for 5 min, the bottom temperature reached 75 °C, while the upper temperature remained at 27 °C (Fig. 6e–f). The upper temperature of the assembly was consistent with that of the ambient temperature. The ambient temperature retention of the multilayered device was comparable to that of a commercial insulation board, indicating that it had remarkable energy-saving benefits (Fig. 6g). As shown in Fig. 6h, compared with the mezzanine building device not covered with ITO film, the infrared emissivity of the multilayered device covered with ITO film dropped from 0.962 to 0.640 at 3 ~ 5 μm, and that of the mezzanine building device decreased from 0.948 to 0.725 at 8 ~ 14 μm. For the bands of these two “atmospheric windows,” the infrared emissivity of the sample surface reduced, indicating that the combination of translucent whole bamboo, transparent bamboo, and ITO film could effectively reduce infrared radiation energy density.

The shielding effectiveness of the multilayered device was further introduced to explore the possibilities of translucent whole bamboo in other fields. The multilayered device with the ITO film exhibited excellent electromagnetic shielding properties. Figure 6i shows that the SE of the multilayered device with a thickness of 105 mm was 46.3 dB at 8.2 ~ 12.4 GHz, which meets the requirement of 20 dB for commercial electromagnetic shielding applications. It is noteworthy that virtually no studies have investigated the electromagnetic shielding properties of transparent biomass materials (Table S3). The shielding efficiency of a concrete wall with a thickness of 300 mm was only 3 ~ 10 dB in the frequency band of 0.03 ~ 1 GHz [67–71]. Notably, the reflectance of the multilayered device was close to 1 (Fig. S8). Owing to the urgent demand for electromagnetic wave radiation protection in civil buildings, radar stations, underground warehouses, airports, and other targets, the reasonable use of composite materials can improve the shielding efficiency of buildings and effectively reduce the damage caused by electromagnetic radiation.

The multilayer device composed of translucent whole bamboo, transparent bamboo, and ITO film has the advantages of good light transmittance, heat insulation, electromagnetic shielding, and so on, which is suitable for energy-saving building materials.

## Conclusions

Thus, we demonstrated that whole bamboo could be converted into a translucent cellulose composite material with good optical properties in a simple, fast, and efficient processing manner without destroying the shape of the whole bamboo. The delignification method using H_2_O_2_ and acetic acid and impregnation with UV resin played a key role. Delignification improved the oxidation performance of H_2_O_2_ in acidic solution, enabling it to quickly penetrate high-density bamboo, remove lignin, and thereby create a porous structure. This delignification method could also be extended to other biomass materials with high densities and low porosities. Impregnation with the UV resin made the bamboo translucent and curable by UV light. The light transmittance of the whole bamboo composite with a wall thickness of 6.23 mm could get as high as 59.2%, with a maximum tensile strength of 46.40 MPa, and surface hardness of up to 80.2 HD. The shielding effectiveness of the multilayered device was 46.8 dB at 8.2 ~ 12.4 GHz. Multilayered devices have great potential in the field of architectural decorative materials, such as interior space partitions, merchandise displays, billboards, lighted ceilings, furniture decoration, and lighting design. The combination of excellent transmittance, mechanical properties, surface properties, thermal performance, and electromagnetic shielding properties makes this composite attractive for applications in transparent, energy-saving, and electromagnetic shielding buildings.

### Supplementary Information

Below is the link to the electronic supplementary material.

Supplementary file1 (MP4 1307 KB)

Supplementary file2 (MP4 1030 KB)

Supplementary file3 (PDF 913 KB)
