# Supplementary material for: Green, Sustainable Architectural Bamboo with High Light Transmission and Excellent Electromagnetic Shielding as a Candidate for Energy-Saving Buildings
Source: Nanomicro Lett. 2022 Dec 10;15:11. doi: 10.1007/s40820-022-00982-7 (PMC9741695; doi:10.1007/s40820-022-00982-7)
Supplement: Supplementary file 3 — Supplementary file3 (PDF 913 KB) [file 40820_2022_982_MOESM3_ESM.pdf]

Supporting Information for

Green, Sustainable Architectural Bamboo with High Light Transmission and Excellent Electromagnetic Shielding As A Candidate For Energy-Saving Buildings

Jing Wang[1, 3], Xinyu Wu[1, 3], Yajing Wang[1, 3], Weiying Zhao[1, 3], Yue Zhao[2], Ming Zhou[2], Yan Wu[1, 3, *], Guangbin Ji[2, *]

[1] College of Furnishings and Industrial Design, Nanjing Forestry University, Nanjing 210037, P. R. China

[2] College of Materials Science and Technology, Nanjing University of Aeronautics and Astronautics, Nanjing 211100, P. R. China

[3] College of Materials Science and Engineering, Nanjing Forestry University, Nanjing, Nanjing 210037, P. R. China

*Corresponding authors. E-mail: wuyan@njfu.edu.cn (Yan Wu), gbji@nuaa.edu.cn (Guangbin Ji)

S1 Supplementary Tables and Figures

Table S1 Naming of the samples

Species	Delignification treatment method	Delignification time (h)
H2	A mixture of hydrogen peroxide and acetic acid	2
H4		4
H6		6
H8		8
H10		10
H12		12
S2	Acid sodium chlorite	2
S4		4
S6		6
S8		8
S10		10
S12		12

Table S2 The curing time, thickness, and shape of our whole bamboo and other biomass transparent materials

Species	Polymer	Curing time/h	Thickness/mm	Shape	Refs.
Whole bamboo	UV resin	0.25	6.23	Round and straight hollow	This work
Balsa wood veneer	PMMA	4	0.8	Tablet	[S1]
Balsa wood film	Epoxy	NA	0.7	Tablet	[S2]

Bamboo strip	Epoxy	24	1	Tablet	[S3]
Pine wood veneer	Epoxy	24	0.6	Tablet	[S4]
Balsa wood veneer	PMMA	4	3.7	Tablet	[S5]
Basswood veneer	Epoxy	12	3	Tablet	[S6]
Basswood veneer	Epoxy	NA	1.4	Tablet	[S7]
Betula alnoides veneer	PMMA	5	0.5	Tablet	[S8]
Oguman veneer	PMMA	5	0.15	Tablet	[S9]
Scotch pine veneer	Epoxy	12	0.2	Tablet	[S10]
Balsa wood veneer	PMMA	6	0.24	Tablet	[S11]
Balsa veneer	PVA	48	0.8	Tablet	[S12]
Fir veneer	Cellulose nanofiber suspensions	48	NA	Tablet	[S13]
Bamboo veneer	Epoxy	24	0.3	Tablet	[S14]
Bamboo veneer	Epoxy	24	0.3	Tablet	[S15]
Bamboo veneer	Epoxy	NA	1.5	Tablet	[S16]

NA: Not available.

Table S3 Tensile strength and electromagnetic shielding properties of our whole bamboo and other biomass transparent materials

**Species**	**Polymer**	**Tensile strength/MPa**	**Electromagnetic shielding/dB**	**Refs.**
Whole bamboo	UV resin	46.4	46.3	This work
Balsa wood film	Epoxy	33.29	NA	[S2]
Basswood veneer	Epoxy	45.38	NA	[S6]
Basswood veneer	Epoxy	≈13	NA	[S7]
Oguman veneer	PMMA	≈35	NA	[S9]
Scotch pine veneer	Epoxy	≈35	NA	[S10]
Bamboo veneer	Epoxy	39.75	NA	[S15]
Balsa wood veneer	Epoxy	46.2	NA	[S17]
Balsa wood slice	DES	1.14	NA	[S18]
Balsa wood veneer	PMMA	≈45	NA	[S19]
Poplar wood slice	PMMA	45.92	NA	[S20]

Cathay poplar slice	PMMA	28.7	NA	[S21]
Paulownia wood veneer	Epoxy	3.74	NA	[S22]
Poplar wood slice	PVA	22.6	NA	[S23]
Balsa wood veneer	Epoxy	9.4	NA	[S24]
Balsa wood veneer	MXene/PVA	≈35	NA	[S25]

NA: Not available.

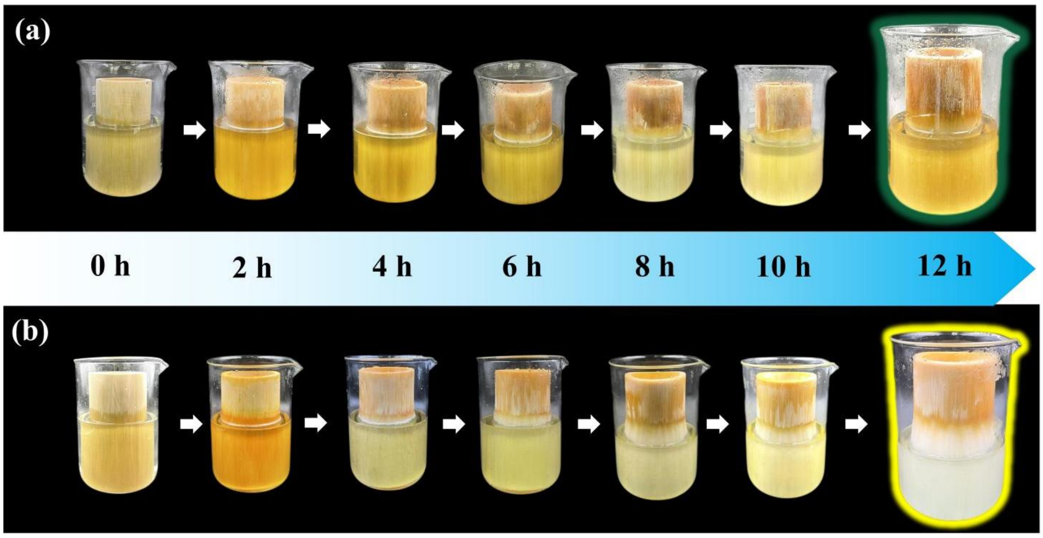

Fig. S1 Variation of whole bamboo with time in different delignification solutions, **a** Variation of whole bamboo in acidic sodium chlorite solution, **b** Variation diagram of whole bamboo in the mixed solution of hydrogen peroxide and acetic acid

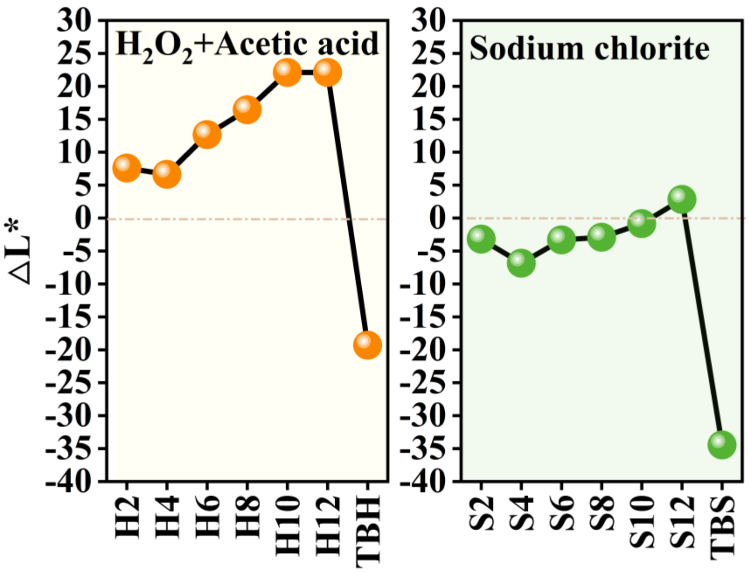

Fig. S2 Brightness difference of samples

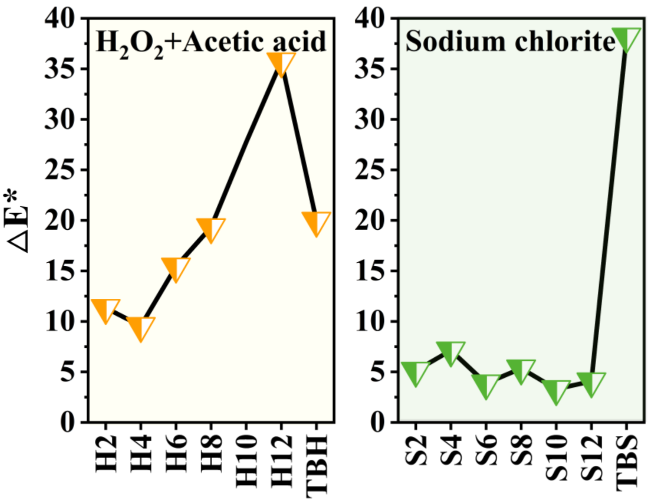

Fig. S3 Total color difference of samples

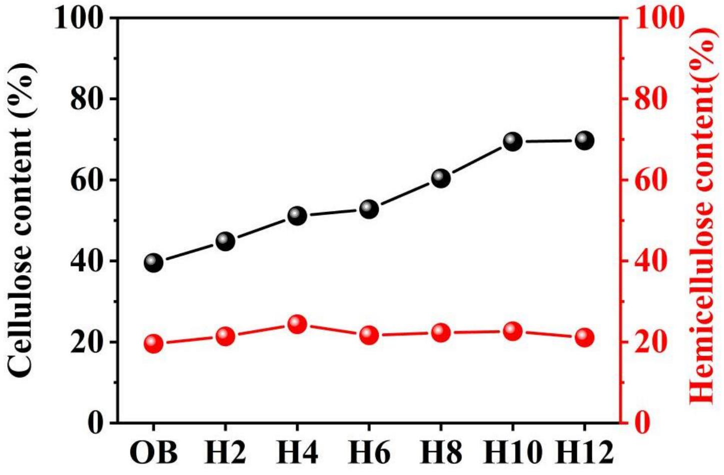

Fig. S4 Relative content of cellulose and hemicellulose of the samples after treatment with hydrogen peroxide and acetic acid

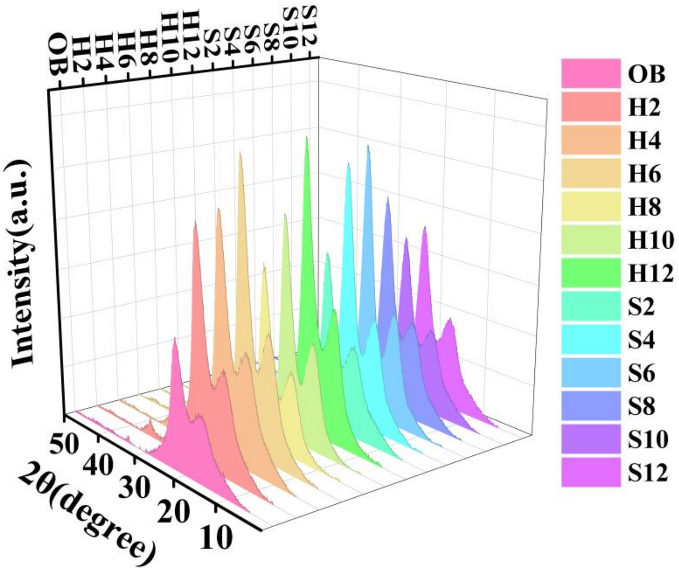

Fig. S5 XRD curves of OB and delignified samples

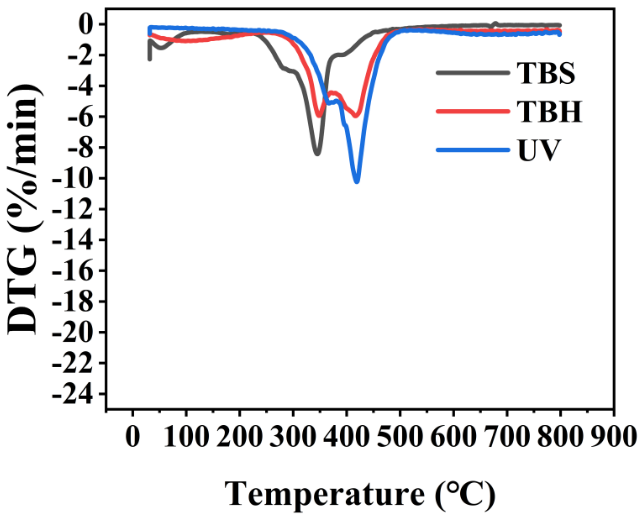

Fig. S6 DTG curves of TBS, TBH and UV

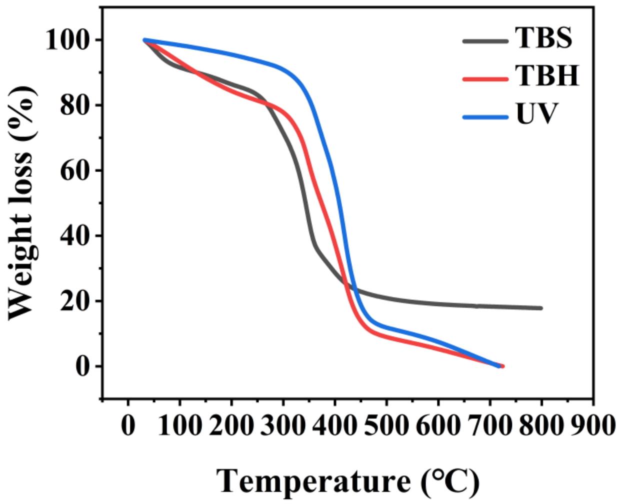

Fig. S7 TG curves of TBS, TBH and UV

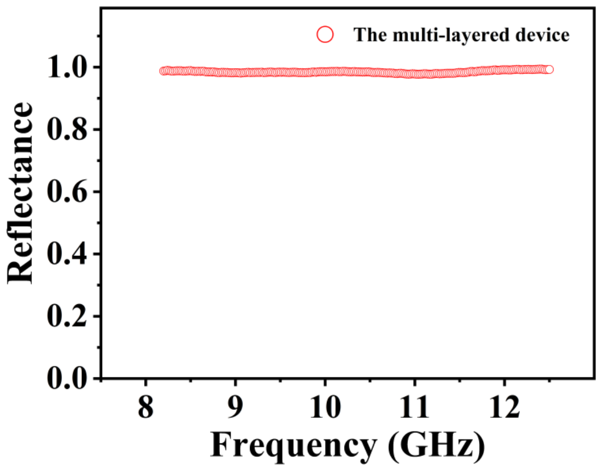

Fig. S8 Reflectance curve of the multi-layered device

S2 Characterization

The chemical groups in all samples were tested and analyzed by Fourier transform infrared spectroscopy (FTIR, ERTEX 80 V, Bruker, Germany). The delignification treatment effect and impregnation effect of different delignification methods were analyzed by observing the change of chemical group vibration peak under light transmission with wavenumbers of 400-4000 cm^{-1} before and after treatment. To determine the crystal structure of the samples before and after delignification treatment, the samples were tested by an X-ray single crystal diffractometer (XRD, Bruker D8 venture, Germany). The test scope of 2θ was 5–50, with an interval equal to 0.02. The relative content of lignin in OB, H2, H4, H6, H8, H10, H12, S2, S4, S6, S8, S10, and S12 was determined by the method of the National Renewable Energy Laboratory. All samples were processed into 60 mesh powder. The cross-section and longitudinal section of the samples were investigated and analyzed by scanning electron microscope (SEM, quanta200, Fei, USA).

The UV Visible photometer (U3900, HITACHI, Japan) was used to measure the optical transmittance of the samples under visible light with wavelengths of 380-780 nm. The transmittance was calculated according to Eq. S1, where T_1 is the incident light and T_2 is the transmission light of the sample.

$$Transmittance = \frac{T_2}{T_1} \times 100\% \tag{S1}$$

The L*a*b coordinate of the samples was recorded by an RM200 color tester. Based on L*a*b of the OB sample, the difference between the values of the sample and OB was recorded as $\Delta L*$. The total color difference ΔE_{ab}^* was calculated according to Eq. S2. $\Delta L*$ represents the change of light and dark degrees of the sample. A positive value indicates a brightening of the sample and a negative value indicates a darkening of the sample. The greater the absolute value of the value, the greater the change.

$$\Delta E_{ab}^* = \sqrt{(\Delta L^*)^2 + (\Delta a)^2 + (\Delta b^*)^2} \tag{S2}$$

TES-1330A digital illuminometer was used to measure the illumination of samples at different distances. Before the illumination test, some small LED bulbs were placed inside each sample.

After splitting the round straight hollow samples into several pieces along the fiber growth direction, longitudinal tensile tests were carried out on the samples using an AG-IC precision electro-mechanical testing machine with a tensile rate of 3 mm min^{-1} and a maximum load force of 10,000 N. The maximum tensile strength of each sample was calculated based on the sector cross-section of each piece with the maximum tensile force. The maximum tensile load that the samples could withstand was recorded. The surface hardness values of the samples were tested and recorded using a LX-D Shao rubber durometer, and the hardness changes of the samples were comparatively analyzed. The thermal degradation of samples from 0-800 °C was tested using a thermogravimetry and synchronous thermal analyzer (TGA, METTLER Telodo TGA/DSC1, Sweden) under a nitrogen atmosphere with a heating rate of 10 °C min^{-1}.

The mass of the samples before and after water absorption was recorded after being placed in distilled water solution under the condition of room temperature (25 °C) for more than 25 h, and the change ratio of the mass was calculated according to Eq. S3, where W is the weight gain rate, M_1 is the weight after absorbing water; M_0 is the weight before absorbing water.

$$W = \frac{M_1 - M_0}{M_0} \tag{S3}$$

The infrared emissivity of the multi-layered device was measured by a dual-band emissivity meter (IR-2, Shanghai Chengbo Optoelectronic Technology Co., LTD) at 3~5 μm and 8~14 μm. After the temperature of the heating gasket was heated to 70 °C, the multi-layered device and

extruded polystyrene thermal insulation (XPS) board are placed on the heating gasket respectively, infrared thermal images of the multi-layered device and XPS board were captured by an infrared camera (FOTRIC 227S). The temperature recorder was used to record the change of the temperature of the uppermost layer of the multi-layered device and XPS board every second within 5 min.

The vector network analyzer N5244A of Agilent and the waveguide method were used to test the shielding effectiveness (*SE*) of the samples in the X band (8.2 GHz ~ 12.4 GHz) at room temperature. The surface reflection (SE_R), internal absorption (SE_A), the total shielding effectiveness (SE_T), reflectance (*R*), transmittance (*T*), and absorbance (*A*) could be described by the following formulas:

$$R = |\mathrm{S}_{11}|^2 = |\mathrm{S}_{22}|^2 \tag{S4}$$

$$T = |\mathrm{S}_{12}|^2 = |\mathrm{S}_{21}|^2 \tag{S5}$$

$$A = 1 - \mathrm{R} - \mathrm{T} \tag{S6}$$

$$SE_R(dB) = -10\log(1 - \mathrm{R}) \tag{S7}$$

$$SE_A(dB) = -10\log(\mathrm{T}/1 - \mathrm{R}) \tag{S8}$$

$$SE_T(\mathrm{dB}) = \mathrm{SE_R} + \mathrm{SE_A} + \mathrm{SE_M} \tag{S9}$$

Herein, SE_M represents the multiple interior reflections, which can be ignored when SE_T>15 dB.

Supplementary References

[S1] Q. Fu, M. Yan, E. Jungstedt, X. Yang, Y. Li et al., Transparent plywood as a load-bearing and luminescent biocomposite. Compos. Sci. Technol. **164**, 296-303 (2018). https://doi.org/10.1016/j.compscitech.2018.06.001

[S2] C. Jia, C. Chen, R. Mi, T. Li, J. Dai et al., Clear wood toward high-performance building materials. ACS Nano **13**(9), 9993-10001 (2019). https://doi.org/10.1021/acsnano.9b00089

[S3] X. Wang, S. Shan, S.Q. Shi, Y. Zhang, L. Cai et al., Optically transparent bamboo with high strength and low thermal conductivity. ACS Appl. Mater. Interfaces **13**(1), 1662-1669 (2021). https://doi.org/10.1021/acsami.0c21245

[S4] R. Mi, C. Chen, T. Keplinger, Y. Pei, S. He et al., Scalable aesthetic transparent wood for energy efficient buildings. Nat. Commun. **11**, 3836 (2020). https://doi.org/10.1038/s41467-020-17513-w

[S5] Y. Li, Q. Fu, S. Yu, M. Yan, L. Berglund, Optically transparent wood from a nanoporous cellulosic template: combining functional and structural performance. Biomacromolecules **17**(4), 1358-1364 (2016). https://doi.org/10.1021/acs.biomac.6b00145

[S6] M. Zhu, J. Song, T. Li, A. Gong, Y. Wang et al., Highly anisotropic, highly transparent wood composites. Adv. Mater. **28**(26), 5181 (2016). https://doi.org/10.1002/adma.201600427

[S7] M. Zhu, T. Li, C.S. Davis, Y. Yao, J. Dai et al., Transparent and haze wood composites for highly efficient broadband light management in solar cells. Nano Energy **26**, 332-339 (2016). https://doi.org/10.1016/j.nanoen.2016.05.020

[S8] Y. Wu, J.C. Zhou, Q.T. Huang, F. Yang, Y.J. Wang et al., Study on the properties of partially transparent wood under different delignification processes. Polymers **12**(3), 661 (2020). https://doi.org/10.3390/polym12030661

[S9] Y. Wu, J.C. Zhou, Q.T. Huang, F. Yang, Y.J. Wang et al., Study on the colorimetry properties of transparent wood prepared from six wood species. ACS Omega **5**(4), 1782-1788 (2020). https://doi.org/10.1021/acsomega.9b02498

[S10] Y.J. Wang, Y. Wu, F. Yang, J. Wang, J.C. Zhou, A multilayer transparent wood prepared by laminating two kinds of tree species. J. Appl. Polym. Sci. **139**(13), e51872 (2022). https://doi.org/10.1002/app.51872

[S11] H. Chen, A. Baitenov, Y. Li, E. Vasileva, S. Popov et al., Thickness dependence of optical transmittance of transparent wood: chemical modification effects. ACS Appl. Mater. Interfaces **11** (38), 35451-35457 (2019). https://doi.org/10.1021/acsami.9b11816

[S12] R. Mi, T. Li, D. Dalgo, C. Chen, Y. Kuang et al., A clear, strong, and thermally insulated transparent wood for energy efficient windows. Adv. Funct. Mater. **30**(1), 1907511 (2020). https://doi.org/10.1002/adfm.201907511

[S13] L.V. Hai, R.M. Muthoka, P.S. Panicker, D.O. Agumba, H.D. Pham et al., All-biobased transparent-wood: a new approach and its environmental-friendly packaging application. Carbohyd. Polym. **264**, 118012 (2021). https://doi.org/10.1016/j.carbpol.2021.118012

[S14] Y. Wu, J. Wang, Y.J. Wang, J.C. Zhou, Properties of multilayer transparent bamboo Materials. ACS Omega **6**(49), 33747-33756 (2021). https://doi.org/10.1021/acsomega.1c05014

[S15] J. Wang, Y.J. Wang, Y. Wu, W.Y. Zhao, A multilayer transparent bamboo with good optical properties and UV shielding prepared by different lamination methods. ACS Sustain. Chem. Eng. **10**(18), 6106-6116 (2022). https://doi.org/10.1021/acssuschemeng.2c01719

[S16] K.L. Wang, H.Z. Peng, Q.Y. Gu, X.Z. Zhang, X.R. Liu et al., Scalable, large-size, and flexible transparent bamboo. Chem. Eng. J. **451**, 138349 (2022). https://doi.org/10.1016/j.cej.2022.138349

[S17] Q.Q. Xia, C.J. Chen, T. Li, S.M. He, J.L. Gao et al., Solar-assisted fabrication of large-scale, patternable transparent wood. Sci. Adv. **7**(5), eabd7342 (2021). https://doi.org/10.1126/sciadv.abd7342

[S18] L.C. Yang, Y. Wu, F. Yang, W.H. Wang, Study on the preparation process and performance of a conductive, flexible, and transparent wood. J. Mater. Res. Technol. **15**, 5396-5404 (2021). https://doi.org/10.1016/j.jmrt.2021.11.021

[S19] Y.Y. Li, M. Cheng, E. Jungstedt, B. Xu, L.C. Sun et al., Optically transparent wood substrate for perovskite solar cells. ACS Sustain. Chem. Eng. **7**(6), 6061-6067 (2019). https://doi.org/10.1021/acssuschemeng.8b06248

[S20] W. Gan, S. Xiao, L. Gao, R. Gao, J. Li et al., Luminescent and transparent wood composites fabricated by poly(methyl methacrylate) and gamma-Fe_2O_3@YVO4:Eu^{3+} nanoparticle impregnation. ACS Sustain. Chem. Eng. **5**(5), 3855-3862 (2017). https://doi.org/10.1021/acssuschemeng.6b02985

[S21] W.T. Gan, L.K. Gao, S.L. Xiao, W.B. Zhang, X.X. Zhan et al., Transparent magnetic wood composites based on immobilizing Fe_3O_4 nanoparticles into a delignified wood

template. J. Mater. Sci. **52**(6), 3321-3329 (2017). https://doi.org/10.1007/s10853-016-0619-8

[S22] H.C. Cai, Z.Q. Wang, D. Xie, P.P. Zhao, J.P. Sun et al., Flexible transparent wood enabled by epoxy resin and ethylene glycol diglycidyl ether. J. Forestry Res. **32**(4), 1779-1787 (2021). https://doi.org/10.1007/s11676-020-01201-y

[S23] A. Rao, G.B. Nagarajappa, S. Nair, A.M. Chathoth, K.K. Pandey, Flexible transparent wood prepared from poplar veneer and polyvinyl alcohol. Compos. Sci. Technol. **182**, 107719 (2019). https://doi.org/10.1016/j.compscitech.2019.107719

[S24] C. Zhang, T. Lin, X.F. Yin, X.Y. Wu, X.Y. Wei, Preparation of transparent wood containing carbon dots for application in the field of white-LED. J. Wood Chem. Technol., (2022). https://doi.org/10.1080/02773813.2022.2085749

[S25] L.R. Zhang, Y.Q. Jiang, L.Y. Zhou, Z.H. Jiang, L. Li et al., Mechanical, thermal stability, and flame retardancy performance of transparent wood composite improved with delaminated $Ti_3C_2T_x$ (MXene) nanosheets. J. Mater. Sci. **57**(5), 3348-3359 (2022). https://doi.org/10.1007/s10853-021-06776-3
